# Gene Silencing of FANCF Potentiates the Sensitivity to Mitoxantrone through Activation of JNK and p38 Signal Pathways in Breast Cancer Cells

**DOI:** 10.1371/journal.pone.0044254

**Published:** 2012-08-28

**Authors:** Yanlin Li, Lin Zhao, Haigang Sun, Jiankun Yu, Na Li, Jingwei Liang, Yan Wang, Miao He, Xuefeng Bai, Zhaojin Yu, Zhihong Zheng, Xiaoyi Mi, Enhua Wang, Minjie Wei

**Affiliations:** 1 Department of Pharmacology, China Medical University, Shenyang City, Liaoning, China; 2 Institute of Pathophysiology, China Medical University, Shenyang City, Liaoning, China; University of Navarra, Spain

## Abstract

Fanconi anemia complementation group-F (FANCF) is a key factor to maintain the function of FA/BRCA, a DNA-damage response pathway. However, the functional role of FANCF in breast cancer has not been elucidated. In this study, we examined the effects and mechanisms of *FANCF*-RNAi on the sensitivity of breast cancer cells to mitoxantrone (MX). *FANCF* silencing by *FANCF*-shRNA blocked functions of FA/BRCA pathway through inhibition of FANCD2 mono-ubiquitination in breast cancer cell lines MCF-7 and T-47D. In addition, *FANCF* shRNA inhibited cell proliferation, induced apoptosis, and chromosome fragmentation in both breast cancer cells. We also found that *FANCF* silencing potentiated the sensitivity to MX in breast cancer cells, accompanying with an increase in intracellular MX accumulation and a decrease in BCRP expression. Furthermore, we found that the blockade of FA/BRCA pathway by *FANCF*-RNAi activated p38 and JNK MAPK signal pathways in response to MX treatment. BCRP expression was restored by p38 inhibitor SB203580, but not by JNK inhibitor SP600125. *FANCF* silencing increased JNK and p38 mediated activation of p53 in MX-treated breast cancer cells, activated the mitochondrial apoptosis pathway. Our findings indicate that *FANCF* shRNA potentiates the sensitivity of breast cancer cells to MX, suggesting that FANCF may be a potential target for therapeutic strategies for the treatment of breast tumors.

## Introduction

Fanconi anemia (FA) is a rare chromosome instability syndrome that is characterized by bone marrow failure, developmental abnormalities, and a high risk for the development of cancers, such as hematological malignancies, solid tumors of the head and neck region, and gynecological tumors [1], [2], [3]. FA has at least 15 fanconi anemia complementation (FANC) groups (FANC A-C, D1, D2, E, F, G, I, J, L, M, N, O, and P) [4], [5], [6]. FA core complex consists of eight proteins (FANCA, B, C, E, F, G, L, and M) and four FA-associated proteins (FAAP24, FAAP100, MHF1 and MHF2), which monoubiquitinate FANCD2 and FANCI. Following monoubiquitination, the FANCD2/I complex is targeted to sites of chromatin damage [7], [8], [9]. FANC proteins are involved in cell cycle regulation, DNA damage and repair, apoptosis, gene transcription, and maintenance of genomic integrity through common FA/ breast cancer susceptibility gene (BRCA) cellular pathways [10].

As an adaptor protein of the FANC group, FANCF stabilizes component of FA core complex, and maintains the biological functions of the FA/BRCA pathway by interacting with the FANCC/FANCE subunit through its N-terminal, and with the FANCA/FANCG subunit through its C-terminal [11]. Epigenetic silencing of *FANCF* has been implicated in ovarian [12], [13], leukemic [14], cervical [15], bladder [16], lung, and oral tumors [17]. Low expression of FANCF is known to lead to FANCD2 ubiquitin inactivation and dysfunction of the FA/BRCA pathway, which promote the sensitivity of tumor cells to DNA cross-linking agents, such as melphalan, cisplatin, and mitomyclin C in gliomas, myelomas, and ovarian cancers[18], [19], [20], [21]. Volinia et al reported that FANCF expression was lost when the ductal breast carcinoma transformed from in situ to invasive one [22]. However, the effect of low expression of FANCF on sensitivity of breast cancer to drugs remains unclear.

A defect in the FA/BRCA pathway induces a hypersensitivity to DNA damaging chemotherapy [16], [23]. However, it remains unknown whether disruption of FA/BRCA pathway is involved in the cytotoxicity of other chemotheraperutic agents such as mitoxantrone. Mitoxantrone (MX), a DNA intercalating agent, exhibits a significant inhibitory effect on topoisomerase II (Topo II), an essential enzyme in DNA synthesis and meiotic division, which is highly expressed in cancer cells [24]. MX is known to stabilize topoisomerase (Topo) II-DNA complexes, to induce crosslinks and double-strand breaks (DSBs) in DNA, and to cause breakdown of the transcription and replication [25]. MX is used routinely in combination with other anticancer drugs as neoadjuvant chemotherapy in the treatment of breast cancers [26], [27], [28]. It is known that a defect in DNA repair underlies the sensitivity of cancer cells to chemotherapeutic drugs. Therefore, we hypothesize that targeting the FA/BRCA pathway to inhibit DNA damage repair via inhibition of FANCF is vital for increasing the sensitivity to MX, the topoisomerase II poison in the breast cancer.

In the present study, we show that specific short hairpin RNA (shRNA) decreases the levels of FANCF, mediates FA/BRCA pathway dysfunction, and potentiates the sensitivity of breast cancer to mitoxantrone through activation of JNK and p38 signal pathways. Our data show that interference of a protein involved in DNA damage repair by using specific shRNA can potentiate the sensitivity of cancer cells to topoisomerase II poisons, suggesting that this can be a new therapeutic approach in cancers.

## Materials and Methods

### Cell Culture

The human breast cancer cell lines T-47D and MCF-7 cells were obtained from the American Type Culture Collection. Adherent cells were maintained in Dulbecco's Modified Eagle Medium (Invitrogen, Carlsbad, CA, USA) containing 10% fetal bovine serum (HyClone, USA), 100U/ml penicillin, and 100 mg/ml streptomycin in a humidified atmosphere with 5% CO_2_ at 37°C.

### Antibodies and reagents

Antibodies against p53, phospho-p53, breast cancer resistance protein (BCRP), multidrug resistance-associated protein (MRP), lung resistance protein (LRP) and P-glycoprotein (P-gp) were from Santa Cruz Biotechnology (Santa Cruz, CA, USA). Antibodies against Jun N-terminal kinase (JNK), phospho-JNK, extracellular signal-regulated kinase (ERK), phospho-ERK, p38, phospho-p38, and β-actin were from Cell Signaling Technology (Beverly, MA, USA). Antibodies against FANCF, FANCD2, cleaved-caspase-9, caspase-3, caspase-6, and poly ADP-ribose polymerase (PARP) were from Abcam Inc (Cambridge, MA, USA). MX, low melting point (LMP) and normal melting point (NMP) agarose, and 3–(4,5-dimethylthiazol-2-yl) -2,5-diphenyltetrazolium bromide (MTT) were purchased from Sigma Chemicals (St. Louis, Mo, USA). The JNK inhibitor SP600125 and the p38 inhibitor SB203580 were from Calbiochem-Merck (Darmstadt, Germany).

### Construction of the *FANCF* shRNA expression vector

The *FANCF* shRNA expression vector was used to achieve specific down-regulation of FANCF. In brief, DNA vectors expressing the shRNA forms were generated using pSilencer^TM^4.1- CMV plasmid. The vector expressing *FANCF* shRNA oligonucleotides (5′-AACTTCCTGAAGGTG ATAGCG-3′) was used predominantly throughout this study. A scrambled shRNA with no significant homology to human gene sequences was used as a negative control to detect nonspecific effects.

### *FANCF* shRNA transfection

Cells were seeded into six-well plates (3×10^5^cells/well) or 100 mm dishes (2×10^6^cells), and were allowed to adhere for 24 hours. After 24 h, cells were transfected with the pSilencer^TM^ 4.1-CMV Control shRNA vector (hereafter, control shRNA) or pSilencer^TM^ 4.1-CMV *FANCF* shRNA vector (hereafter, *FANCF* shRNA) using Lipofectamine 2000 (Invitrogen, Carlsbad, CA) according to the manufacturer's instructions. After 4 h, the culture medium was replaced with fresh media supplemented with 10% FBS, and the cells were harvested at 24 and 48 h after transfection. Transfection efficiency was observed by fluorescence microscopy and flow cytometry after 48 h, and was as high as 45–55%.

### Western blot analysis

Western analysis for the presence of specific proteins or phosphorylated forms of proteins was performed on whole-cell sonicates and lysates from T-47D and MCF-7 cells. Protein (30∼50 ug) was mixed 4∶1 with 5×sample buffer (20% glycerol, 4% sodium dodecyl sulfate, 10% β-mercaptoethanol, 0.05% bromophenol blue, and 1.25 M Tris–HCl, pH 6.8; all from Sigma). Equal amount of proteins were loaded onto a 10% sodium dodecyl sulfate–polyacrylamide gel. Cell proteins were transferred to PVDF membranes. The PVDF membranes were blocked with 5% milk in Tris-buffered saline with 0.1% Tween 20, and then incubated with an appropriate dilution of antibodies (1∶1,000 to 1∶2,000) overnight at 4°C. The blots were washed and incubated for 1 h with horseradish peroxidase-conjugated anti-IgG antibodies (Santa Cruz Biotechnology). Immunocomplexes were visualized by chemiluminescence using ECL (Santa Cruz Biotechnology).

### Immunodetection of FANCD2 foci

MCF-7 and T-47D breast cancer cells were plated on glass cover slips at 50% conﬂuence and then 16 h later were exposed to *FANCF* shRNA or left untreated. At 24, and 48 h following exposure, cells were washed with PBS, permeabilized with ice-cold 0.5% Triton X-100 in PBS, and then fixed with 2% paraformaldehyde and blocked with 5% bovine serum albumin at room temperature. FANCD2 was detected by incubation with anti-FANCD2 antibody (1∶500) for 90 min at room temperature and then with goat anti-rabbit antibody–Alexa- 488 (1∶1,000; Invitrogen). All slides were counterstained with DAPI and visualized by fluorescence microscopy. The experiment was done in triplicate.

### Cell viability assay

Loss of cell viability was measured by the MTT assay. Cells were seeded at 1×10^4^ cells/well in 96-well plates, and allowed to grow in the growth medium for 24 hours. Cells were transfected with control or *FANCF* shRNA for 48 h, and then treated with MX (10 μM) for 24 h. After the drug treatment, cells were incubated with 5 mg/ml MTT for 4 hours, and subsequently solubilized in DMSO (100 µl/well). The absorbance at 570 nm was then measured using an ELISA reader. Experiments were repeated at least three times, and the data was expressed as the means ± SD.

### Flow cytometry

Flow cytometry analysis was performed on a FACS Calibur (Becton-Dickinson). The cationic fluorescent carbocyaninedye, 5,5′,6,6′- tetrachloro -1,1′,3,3′- tetraethylbenzimidazolylcarbocyanine iodide (JC-1) was used to assess changes in the mitochondrial membrane potential observed in apoptotic cells. Cells were incubated for 15 min at 37°C with 15 µg/ml JC-1 before analysis [29]. For detection of apoptotic cells, cells were harvested, washed twice with PBS, then incubated for 15 min at room temperature with a solution of fluorescence isothiocyanate (FITC) conjugated annexin V (2.5 µg/ml) and PI (5 µg/ml) (all from Sigma), and analyzed for apoptosis. For determination of MX accumulation, drug accumulation assay was performed as described previously [30] with some modifications. Briefly, MX was added to cells to a final concentration of 10 μM. The cells were incubated for 24 h at 37°C with 5% CO_2_ in the darkness. After the influx step, the cells were washed with ice-cold phosphate-buffered saline (PBS). The analysis was performed in flow cytometer.

### DNA fragmentation assays

The alkaline comet assay was performed essentially according to the procedure of Singh et al. with modifications as described previously [31], [32]. A freshly prepared suspension of cells in 1% LMP agarose dissolved in PBS was spread onto microscope slides precoated with 0.6% NMP agarose. The cells were then lysed for 1 h at 4°C in a buffer consisting of 2.5 M NaCl, 100 mM EDTA, 1% Triton X-100, 10 mM Tris, pH 10. After lysis, the slides were placed in an electrophoresis unit, and the DNA was allowed to unwind for 40 min in the electrophoretic solution consisting of 300 mM NaOH, 1 mM EDTA, pH>13. Electrophoresis was conducted at 4°C (the temperature of the running buffer did not exceeded 12°C) for 20 min at 25V and 300 mA. The slides were then neutralized with 0.4 M Tris, pH 7.5, stained with 2.5 mM PI and covered with cover slips. To prevent additional DNA damage, all the steps described above were conducted under dimmed light or in the dark. Five hundred randomly chosen cells per slide were scanned and analyzed automatically using casp1.01 software. Mean tail length was calculated for∼400 cells.

### Statistical analysis

Data are represented as the mean ± the standard deviation (SD). Data were analyzed using the one-way ANOVA with post-hoc analysis. *P*<0.05 was considered statistically significant.

## Results

### Gene silencing of *FANCF* blocks function of FA/BRCA

**Figure 1:**
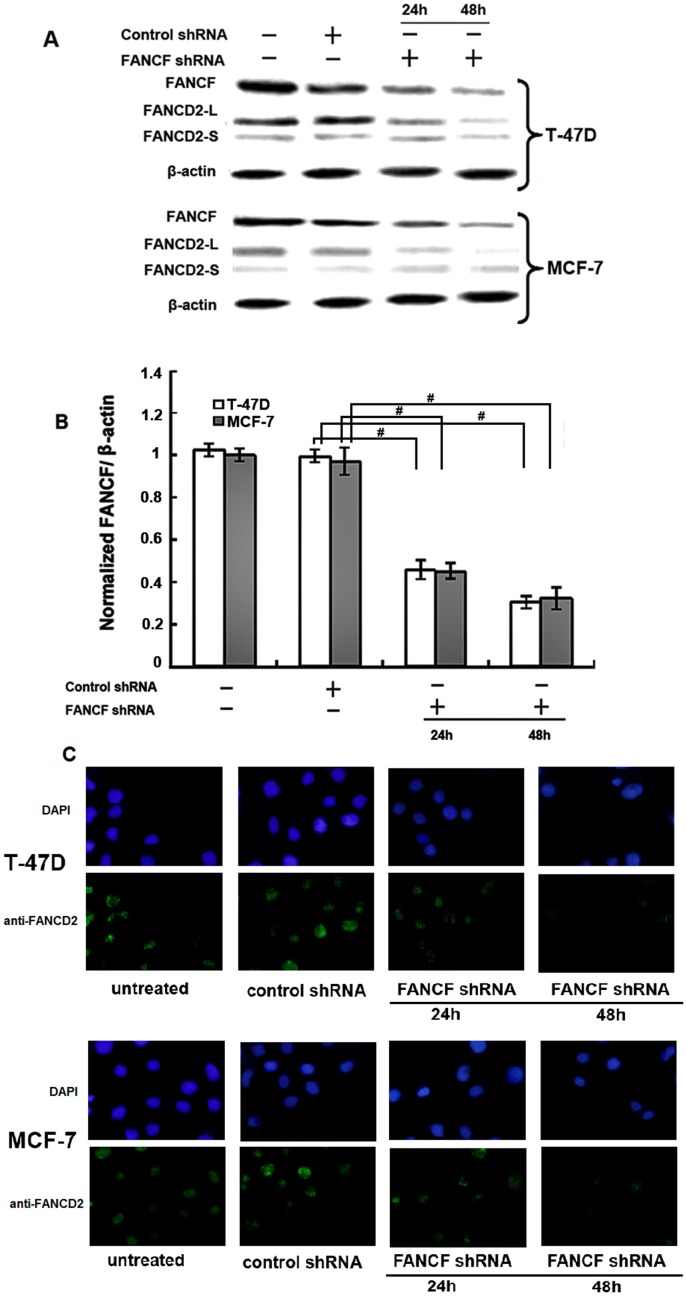
Inhibition of FANCF levels, FANCD2 ubiquitination and foci formation by *FANCF* shRNA in breast cancer cell lines. MCF-7 and T-47D cells were transfected with *FANCF* shRNA and control shRNA(scrambled shRNA) for 24 h and 48 h, then protein was extracted for western blotting with anti-FANCF and anti-FANCD2 antibodies (FANCD2-L = mono-ubiquitinated; FANCD2-S  = nonubiquit- inated). β-actin was simultaneously immunodetected to verify equal loading of cell lysates. (A) Representative FANCF and FANCD2 blots. Three independent experiments were performed. (B) Densitometric analysis was done for FANCF expression. Results were normalized to β-actin values. Graphs show means ± S.D. of three independent experiments. *P* values, ^#^ *P*<0.05. (C) FANCD2 foci formation was detected by immunofluorescence. Representative images are shown.

Human breast cancer cell lines, MCF-7 and T-47D, were used in this study. Their molecular characteristics are listed in Table 1. ShRNA was used to knock-down *FANCF* expression in MCF-7 and T-47D breast cancer cells. To verify the results of gene silencing, FANCF expression was detected by western-blotting at 24 h and 48 h post-transfection. We found that expression of FANCF in the two cell lines (MCF-7 and T-47D) was inhibited in a time-dependent manner, as compared with the control (cells treated with scrambled shRNA) (Fig. 1A and Fig. 1B). The results confirmed that FANCF expression was inhibited by transfection with shRNA targeting *FANCF*. Gene silencing of *FANCF* also decreased the expression of FANCD2-L, reduced the level of FANCD2 mono-ubiquitination (Fig. 1A), resulting in the loss of FANCD2 foci (Fig. 1C), However, the expression of FANCD2-S was not reduced in comparison to FANCD2-L in MCF-7 and T-47D cells, although the total amount of FANCD2 appeared slightly reduced. These results suggested that inactivation of the FA/BRCA signaling pathway was induced by gene silencing of *FANCF* in breast cancer cells.

**Table 1:** Characteristics of breast cancer cell lines.

							Topo-II level (absorbance)^a^	
cell ine	ER	p53	Caspase-3	PR	Bcl-2	Her-2 level	*α*	*β*
MCF-7	+	wt	−	+	+	Very low	1086	1233
T-47D	+	mut	+	+	−	Moderate	588	1188

Abbreviation:ER = oestrogen receptor. ^a^Houlbrook *et al* [53].

**Figure 2:**
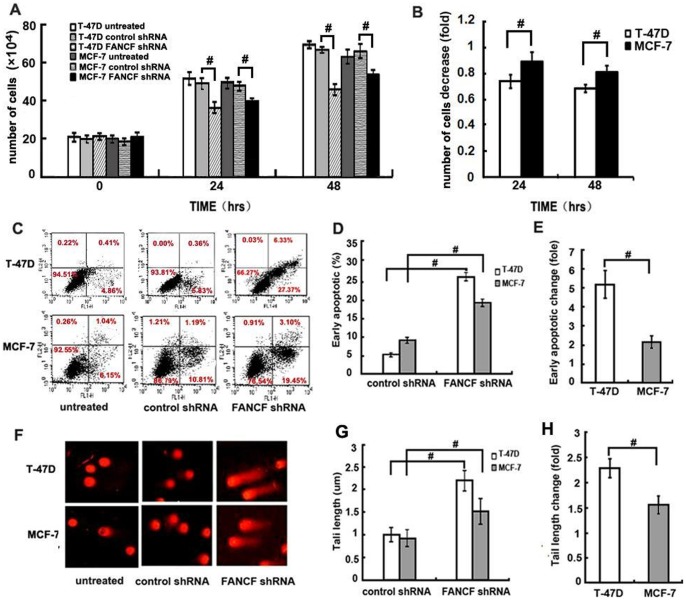
*FANCF* shRNA inhibits cell proliferation, increases apoptosis in T-47D and MCF-7 cells. (A) Number of viable cells was determined using a hemacytometer after staining dead cells with Trypan Blue. (B) Quantitative analysis of the fold decrease of total cell number in MCF-7 cells and T-47D cells treated with *FANCF* shRNA compared with control shRNA. (C) Apoptosis of cells were measured using FACScan after staining with FITC-annexin V and PI. Cells in the lower right-hand quadrant are early apoptotic cells with exposed phosphatidylserine (FITC-annexin V-positive), but intact membrane (PI-negative). (D) The quantification of apoptosis in the indicated cell lines. (F) Single-cell gel electrophoresis (comet assay) showed detectable comet tails when visualized under a fluorescent microscope, indicative of DNA damage. (G) The quantification of DNA fragmentation in the indicated cell lines (control shRNA treated cells was defined as 1.0). (E) and (H) are the quantitative analysis of the fold increase of early apoptotic or tail length in *FANCF-*silenced cells compared with the controls. *P* values, ^#^ *P*<0.05.

*FANCF* shRNA significantly reduced cell proliferation in both cell lines in a time-dependent manner (Fig. 2A). Compared with the control, *FANCF* shRNA induced a decrease in total cell numbers at 24 h and 48 h postransfection in both cell lines. *FANCF* shRNA decreased the total cell number to 89.13%±7.05% of the control at 24 h posttransfection and 81.27%±2.36% of the control at 48 h posttransfection in MCF-7 cell line, which are significantly less than 74.57%±5.19% of the control at 24 h posttransfection and 69.35%±3.31% at 48h posttransfection in T-47D cell line, respectively (*P*<0.05, Fig. 2A), suggesting that *FANCF* shRNA inhibited cell proliferation in T-47D cells more strongly than in MCF-7 cells (*P*<0.05, Fig. 2B).

Next, we investigated the biologic effects (apoptosis, and DNA damage) of *FANCF* silencing at 48h after transfection. After *FANCF* silencing, about 27.37% of MCF-7 cells and 19.45% of T-47D cells underwent apoptosis (Fig. 2C). Compared with the controls, the percentage of apoptotic cells induced by *FANCF* shRNA increased by 2.15±0.32 fold in MCF-7 cells, and by 5.21±0.72 fold in T-47D cells (*P*<0.05, Fig. 2D and Fig. 2E). *FANCF* shRNA induced more apoptotic cells in T-47-D cells than in MCF-7 cells.

We then assessed the effects of *FANCF* silence on DNA damage using the alkaline COMET assay. Inhibition of *FANCF* in both cell types led to a significant increase in DNA damage as measured by tail length in the COMET analysis (Fig. 2F and Fig. 2G). *FANCF* shRNA increased the tail length by 1.55±0.18 fold of the control in MCF-7 cells, which was significantly less than that (2.28±0.19 fold of the control) in T-47D cells (*P*<0.05, Fig. 2H).

### Gene silencing of *FANCF* sensitizes breast cancer cells to MX

**Figure 3:**
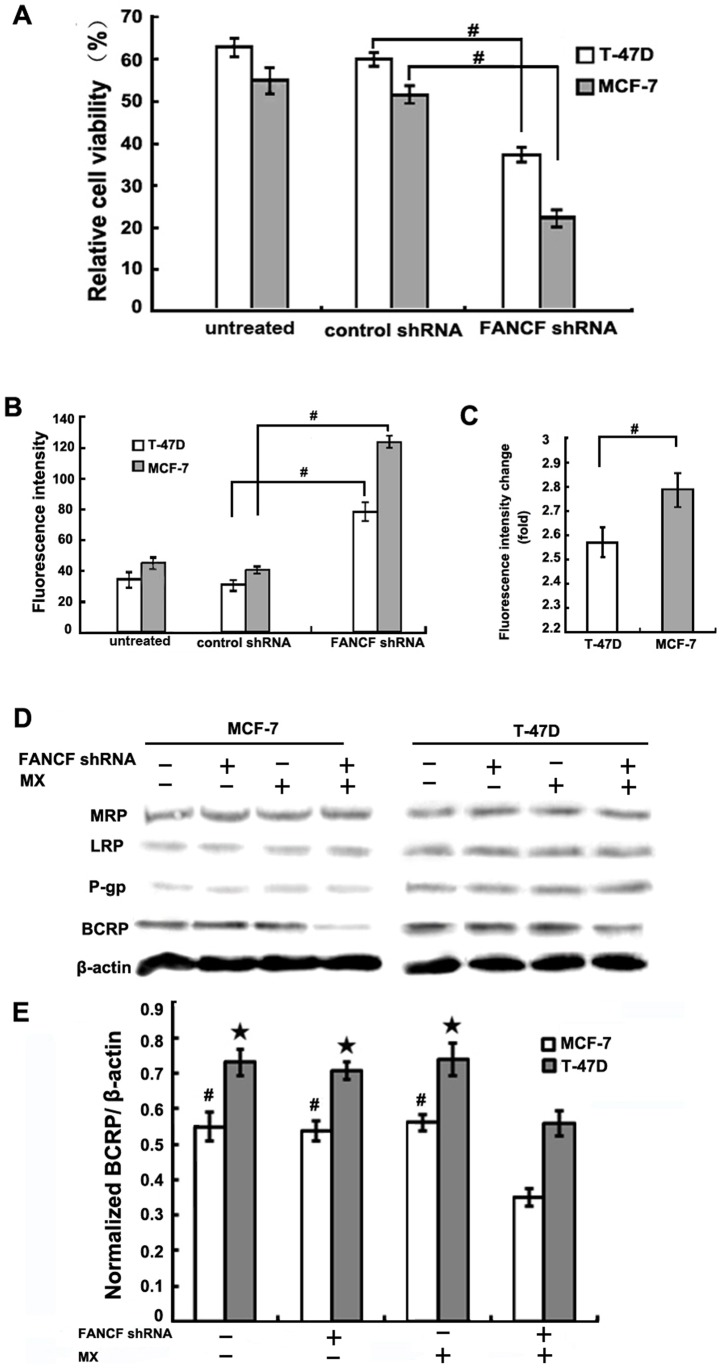
*FANCF* knockdown sensitizes breast cancer cells to MX. (A) T-47D and MCF-7 cells were transfected with *FANCF* shRNA or control shRNA for 48 h, and then treated with 10 uM MX for 24 h. The cell viability was determined by the MTT assay. The percentage of viable cells was determined by the ratio of viable cells treated with MX to that with no MX treatment. (B) Median fluorescene intensity was measured indicating the relative amount of MX accumulation. (C) Quantitative analysis of the fold increase of fluorescence intensity in *FANCF-*silenced MCF-7 and T-47D cells compared with the controls. *P* values, ^#^ *P*<0.05. (D) MRP/LRP/P-gp/ BCRP protein expression was detected by western blot assay. (E) Densitometric analysis was done for BCRP expression. *P* values, ^#^ *P*<0.05 versus transfected with *FANCF* shRNA and MX in MCF-7 cells,★*P*<0.05 versus transfected with *FANCF* shRNA and MX in T-47D cells.

To determine whether *FANCF* silencing affects the sensitivity of breast cancer to MX, antiproliferative actions of MX (10 μM) was evaluated in *FANCF-*silenced MCF-7 and T-47D cells. In the control group, MCF-7 cells and T-47D cells (normal FANCF expression) treated with 10 μM MX exhibited cell viability rates of 51.37%±2.16% and 59.73%±4.14%, respectively. The MX-induced inhibition of cell proliferation in MCF-7 cells was much higher than in T-47D cells (*P*<0.05), suggesting that MCF-7 cells were more sensitive to MX than the T-47D cells. Compared with the control, *FANCF* shRNA significantly enhanced the MX-induced decrease in the cell viability in both cell lines (*P*<0.05, Fig. 3A), suggesting that known-down of *FANCF* significantly potentiated the cytotoxic effects of MX on breast cancers. In addition, *FANCF* shRNA decreased cell viability rate to 21.83%±3.12% of the control in MCF-7 cells, which was significantly less than that 37.03%±4.06% of the control in T-47D cells (Fig. 3A).

We next examined the effects of *FANCF* silencing on MX accumulation in breast cancer cells. After 24 h treatment of 10 μM MX, the amount of MX accumulation in both cell lines increased remarkably in the *FANCF*-silenced cells compared to that in the control cells (*P*<0.05, Fig. 3B). The *FANCF* shRNA-induced increase in MX accumulation in MCF-7 cells (2.76±0.08 fold of the control) was significantly greater than that in T-47D cells (2.57±0.06 fold of the control) (*P*<0.05, Fig. 3C). These results further suggested that *FANCF* silencing potentiated the effects of MX through increasing MX accumulation.

### *FANCF* knockdown downregulates BCRP expression

Overexpression of the ATP-binding cassette transporter (ABC transporter) proteins such as MRP, P-gp, BCRP, and LRP in the cell membrane is known to promote active transport MX out of the cells. These processes decrease intracellular drug concentrations, and lead to multidrug resistance (MDR) of breast cancer cells. Therefore, expression levels of these proteins are considered a useful clinical indicator of tumor cells' drug sensitivity and patient prognosis [33]. Since we observed that *FANCF* gene silencing increased intracellular MX accumulation, we sought to determine whether any of these resistance-related proteins were involved in the *FANCF* silencing-induced effects on MCF-7 and T-47D cells. The results showed that prior to *FANCF* gene silencing, the expression of resistance proteins in T-47D cells was higher than that in MCF-7 cells (Fig. 3D), in agreement with the finding that MX sensitivity of MCF-7 cells was also stronger than that of T-47D cells (Fig. 3A). However, after *FANCF* shRNA and MX combination treatment, BCRP expression was decreased in both cell lines, compared with *FANCF* shRNA or MX treatment alone (Fig. 3D and Fig. 3E). However, there was no significant decrease in BCRP expression in both cell lines treated with MX alone. The MRP, LRP, and P-gp protein expression were not changed under treatment with *FANCF* shRNA and MX. These results suggested that specific inhibition of BCRP expression after *FANCF* silencing contributed to MX accumulation and increased drug sensitivity. BCRP expression in the MCF-7 cells was significantly lower than that in the T-47D cells, and this was consistent with greater amount of intracellular MX accumulation in MCF-7 cells.

### p38 pathway activation accounts for the decreased BCRP expression induced by gene silencing of *FANCF*

**Figure 4:**
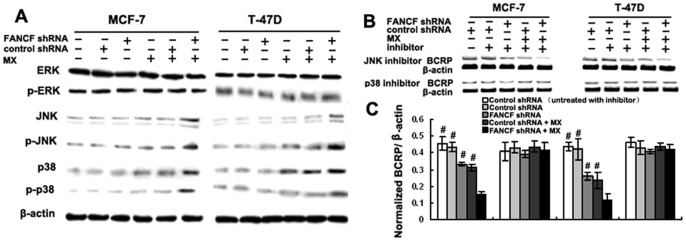
Cotreatment with *FANCF* shRNA and MX inhibits BCRP expression by activating the p38 MAPK pathway. (A) Total cellular proteins (50 μg) from exponentially growing cells treated as indicated in the figure were subjected to western blot analysis with antibodies directed against the proteins or their phosphorylated form as indicated. β-actin was applied as control for equal loading. (B) MCF-7 and T-47D cells were pre-treated with the JNK inhibitor SP600125 or p38 inhibitor SB203580 for 2 h, and then whole lysates from cells treated as indicated in figure were subjected to western blot analysis with BCRP antibody. (C) Densitometric analysis was done for BCRP expression. Results were normalized to β-actin values. Graphs show means ± S.D. of three independent experiments. *P* values, ^#^ *P*<0.05 versus transfected with *FANCF* shRNA and MX in cells.

In the previous experiments, we found that dysfunction of the FA/BRCA pathway induced by *FANCF* gene silencing led to an increased MX sensitivity. These changes appeared to be due to inhibition of BCRP expression. Dysfunction of the FA/BRCA signaling pathway is known to cause activation of the MAPK signaling pathway [34]. Therefore, we examined changes in the expression of proteins association with the MAPK pathway, including JNK, ERK, and p38 in breast cancer cells following *FANCF* gene silencing. The results showed that gene silencing of *FANCF* in MCF-7 and T-47D cells did not affect the protein expression of JNK, ERK, or p38, indicating that *FANCF* silencing in breast cancer cells did not activate the MAPK pathway. However, after treatment with 10 μM MX, which can activate the FA/BRCA pathway, *FANCF*-silenced MCF-7 and T-47D cells, increased the expression of JNK and p38 and the phosphorylation levels of those two proteins. Moreover, the effects were notably higher than those treated with MX alone. ERK expression levels, on the other hand, were unaffected by MX treatment in any of the cell types (Fig. 4A). These results demonstrated that gene silencing of *FANCF* activated JNK and p38 of the MAPK signaling pathway in response to MX treatment.

To further test whether activation of JNK and p38 pathways was involved in the *FANCF* silencing-induced inhibition of BCRP expression, MCF-7 and T-47D cells were treated with the p38 inhibitor, SB203580, and the JNK inhibitor, SP600125. Treated with SB203580, SP600125 alone had no detectable effect on BCRP expression in MCF-7 and T-47D cells. After treatment with SB203580, *FANCF* shRNA had no detectable inhibitory effect on BCRP expression. However, BCRP expression was still inhibited by *FANCF* shRNA in SP600125 treated cells (Fig. 4B and Fig. 4C). These results demonstrated that *FANCF* gene silencing inhibited the expression of BCRP through activation of p38, not JNK pathway.

### *FANCF* gene silencing increases JNK-mediated activation of p53 in MCF-7 and T-47D cells

**Figure 5:**
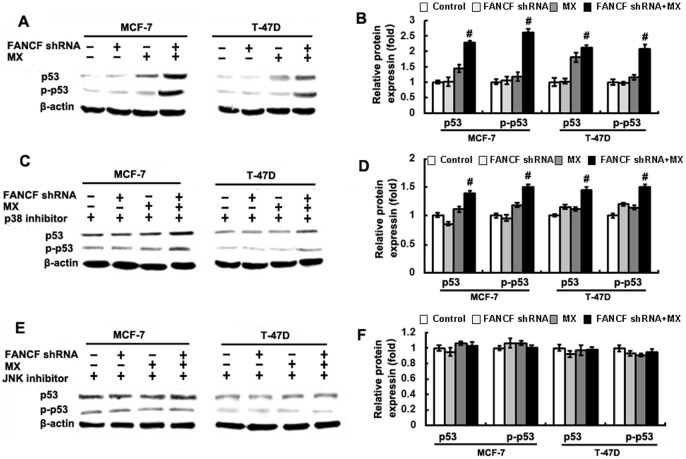
Up-regulation of p53 by *FANCF* shRNA and MX in breast cancer cells. (A) Total cellular proteins (50 μg) from exponentially growing cells treated as indicated in the figure were subjected to western blot analysis with antibodies directed against p53 or p-p53 form as indicated. β-actin was applied as control for equal loading. Cells were pre-treated with p38 inhibitor SB203580 (C) or JNK inhibitor SP600125 (E) for 2 h, and then treated as indicated in the figure were subjected to western blot analysis. (B), (D) and (F) The quantitative analysis of the fold increase of p53 expression in MCF-7 and T-47D cells compared with the controls. *P* values, ^#^ *P*<0.05 versus transfected with *FANCF* shRNA and MX in cells. *P* values, ^#^ *P*<0.05.

We found that MCF-7 and T-47D cell showed different sensitivity to MX after *FANCF* silencing. As shown in Table 1, one of different characteristic between MCF-7 and T-47D cells is p53 status. In addition, JNK activation induces p53 expression [35]. We therefore performed experiments to determine whether *FANCF* silencing had an effect on p53 expression. MX treatment increased phosphorylation levels of p53 in MCF-7 and T-47D cells (Fig. 5A and Fig. 5B). However, compared to MX treatment alone, *FANCF* shRNA and MX treatment increased phosphorylated p53 by 2.2±0.11 fold in MCF-7 cells and by 1.8±0.17 fold in T-47D cells (*P*<0.05, Fig. 5B). p38 inhibitor SB203580 reduced the increase in the expression of phosphorylated p53 by 1.27±0.13 fold in MCF-7 cells and by 1.32±0.27-fold in T-47D cells, compared with controls with no SB203580 treatment (*P*<0.05, Fig. 5C and Fig. 5D), suggesting that p38 inhibitor partly blocked the effect of *FANCF* shRNA on p53. The increase in the expression of phosphorylated p53 was completely blocked by JNK inhibitor SP600125 (Fig. 5D and Fig. 5E). These results suggested that *FANCF* knockdown increased the activation of p53 expression mainly through JNK pathway, partly through p38 pathway in MX-treated MCF-7 and T-47D breast cancer cells.

### *FANCF* gene silencing induced the mitochondrial apoptosis pathway in MCF-7 and T-47D cells

**Figure 6:**
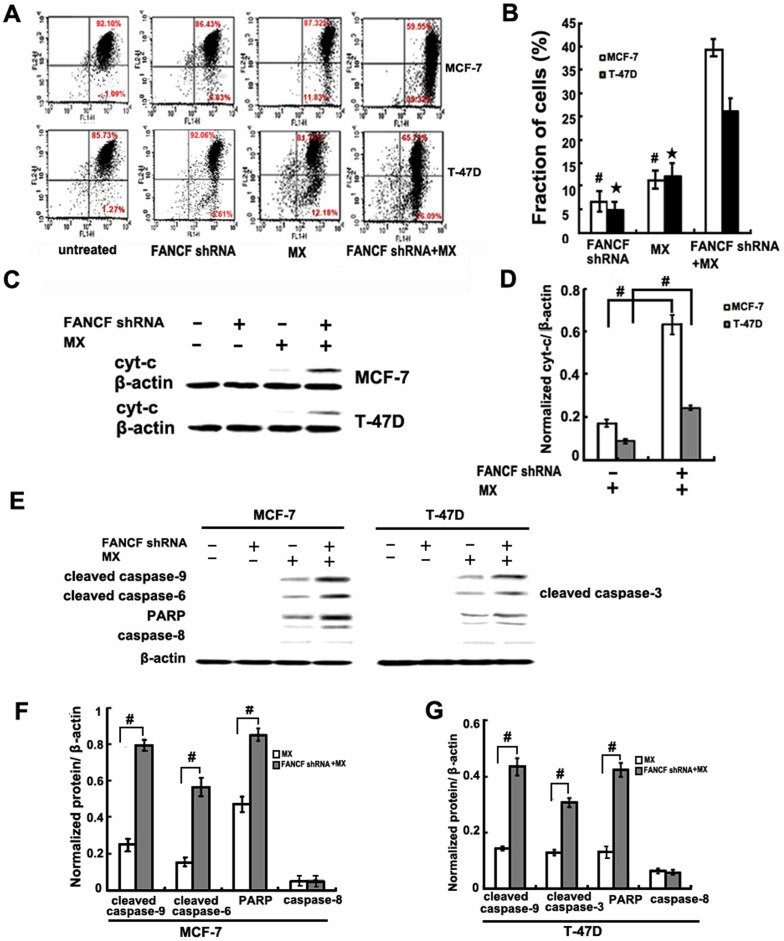
*FANCF* gene silencing induced the mitochondrial apoptosis pathway in MCF-7 and T-47D cells. (A) The cells were stained with JC-1 ﬂuorescence dye, and the change in ΔΨm was examined by FACS. (B) Densitometric analysis was done for fraction of cells. *P* values, ^#^ *P*<0.05 versus transfected with *FANCF* shRNA and MX in MCF-7 cells.★*P*<0.05 versus transfected with *FANCF* shRNA and MX in T-47D cells, (C) Cytosolic lysates were prepared and subjected to SDS-PAGE followed by Western blotting with cyt-c antibody. (D) Densitometric analysis was done for cyt-c expression. *P* values, ^#^ *P*<0.05. (E) Whole lysates from cells treated as indicated in the figure were subjected to western blot analysis with the indicated antibodies recognizing cleaved products of caspase 9, caspase 3, caspase 6 and PARP. (F) Densitometric analysis was done for proteins expression. *P* values, ^#^ *P*<0.05.

Previous studies have shown that activation of the JNK pathway can induce apoptosis [36]. In addition, apoptotic events are different in MCF-7 and T-47D cells [37]. Therefore, changes in the apoptosis pathway were investigated. Disruption of mitochondrial membrane potential (ΔΨm) integrity is one of the early events leading to apoptosis. To assess whether *FANCF* silencing affects the function of mitochondria, ΔΨm changes were measured by employing a mitochondrial fluorescent dye, JC-1. *FANCF* silencing in MX-treated MCF-7 and T-47D cells resulted a decrease in ΔΨm, compared to controls or MX treated alone. Moreover, the *FANCF*-silenced MCF-7 cells had significantly more decrease in the ΔΨm than the *FANCF*-silenced T-47D cells (Fig. 6A and Fig. 6B).

Cytochrome c (cyt-c) release from mitochondria is a critical step in the apoptotic cascade since this activates downstream caspases. Cyt-c was not expressed in the cytoplasm of *FANCF*-silenced MCF-7 or T-47D cells. MX treatment of these cells led to cyt-c expression (Fig. 6C). Compared with MX treatment alone, *FANCF* shRNA and MX treatment increased cyt-c expression by 3.67±0.13 fold in MCF-7 cells and by 2.75±0.34 fold in T-47D cells (Fig. 6D). These findings indicated that the amount of cyt-c in cytoplasm increased as a result of mitochondrial release. Caspase-3/6, caspase-9, PARP cleavage (a substrate of caspase-3), but not caspase-8, were induced by MX treatment in *FANCF*-silenced MCF-7 and T-47D cells (Fig. 6E,). Because caspase-3 was not expressed by the MCF-7 cells, caspase 6 was detected instead and found to be activated [38]. However, compared with MX treatment alone, *FANCF* shRNA and MX treatment increased capase-9, and caspase-6 and PARP by 4.31±0.38 fold, 3.65±0.31 fold, and 2.53±0.12 fold in MCF-7 cells, respectively, and by 3.02±0.05 fold, 2.39±0.17 fold, and 2.24±0.2 fold in T-47D cells, respectively (Fig. 6F and Fig. 6G). The activation of caspase 9 and caspase-6 was stronger in the *FANCF*-silenced MCF-7 cells. These results suggested that *FANCF* silencing induced apoptosis via the mitochondrial apoptosis pathway.

## Discussion

In our present study, gene silencing of *FANCF* in MCF-7 and T-47D breast cancer cells blocked the FA/BRCA pathway as evidenced by reducing the level of FANCD2 mono-ubiquitination, the loss of FANCD2 foci, inhibiting cell proliferation, promoting apoptosis and DNA damage (Fig. 1 and Fig. 2). This is the first study to report on RNAi-mediated *FANCF* silencing-induced dysfunction of the FA/BRCA pathway in breast cancers. Our study also shows that *FANCF* silencing sensitizes breast cancer cells to MX, the topoisomerase II poison-MX. Kachnic et al have reported that FANCD2 promotes cellular resistance to topoisomerase II poisons- etoposide[39]. Taken together, FA/BRCA pathway might play an important role in regulating tumors' sensitivity to many topoisomerase II poisons. Although our studies have demonstrated that *FANCF* silencing significantly increases MX sensitivity *in vitro*, it is critical to further define the role of FANCF in regulating MX sensitivity of breast cancer *in vivo* to evaluate the clinical significance of FANCF. Previous studies show that expression of FANCF is reduced in various human tumors, our results imply the patient with low expression of FANCF might have higher sensitivity to chemotherapy drugs in breast cancers, FANCF may therefore prove to be a novel biomarker for sensitivity to MX-based chemotherapy in breast cancers.

We also find that *FANCF* knockdown by RNAi potentiates MX sensitivity due to selective inhibition of BCRP expression and enhancement of the intracellular drug accumulation. To date, no evidence has been reported in the literature about the regulation of BCRP expression by FANCF. Briot et al found that deficits of FA/BRCA pathway activated the MAPK pathway in FA, thereby increasing TNF-α secretion [34]. Evseenko et al also found that an increase in TNF-α was correlated with a significant decrease in BCRP expression and activity in primary trophoblast cells [40]. Though these studies suggest that the FA/BRCA signaling pathway regulates BCRP expression through the MAPK pathway, no direct experimental evidence has been reported to demonstrate the role of p38 MAPK in the regulation of BCRP by FA/BCRP signaling pathway. Our study shows that *FANCF* silencing in MCF-7 and T-47D breast cancer cells increases MX sensitivity by selectively inhibiting BCRP expression through activation of p38 MAPK pathway (Fig. 4). This is the first report to demonstrate that FANCF is involved in regulating the expression and function of BCRP.

In the present study, we found that p53 activation was absent in FANCF silenced cells without MX treatment (Fig. 5A), although cells already experiencing apoptosis and DNA fragmentation (Fig. 2C, 2F). The reason may be that FA/BRCA pathway dysfunction caused by FANCF silencing, lead to reduction of DNA repair and DNA instability, then induce apoptosis and DNA fragmentation of tumor cells, although absence of p53 activation. However, we also found that p53 is activated in cells treated with MX, the activation is further increased when combined with *FANCF* silencing (Fig. 5A). In addition, we all know that JNK is activated by many cellular stresses, including ultraviolet irradiation, oxidative stresses, inflammatory cytokines, and DNA-damaging agents (cisplatin, etoposide, doxorubicin and MX) [41], [42]. p53 is a downstream factor of the MAPK pathway, and can be activated by JNK [35]. Our study shows that MX activates JNK, and this activation is indispensable for *FANCF* shRNA-induced p53 activation.

Apoptosis induced by the external death receptor pathway or the intrinsic mitochondrial pathway [43]. Both of these two pathways eventually activate caspase 3/6, which are key enzymes of the caspase family of apoptosis proteins. However, activation of caspase 3/6 in the two apoptotic pathways is different. In the death receptor pathway, caspase 3/6 is activated by caspase 8, and in the mitochondrial pathway activation, capase 3/6 activation is mediated via cyt-c/caspase 9 [44]. In the present study, caspase 9 is activated in *FANCF*-silenced MCF-7 and T-47D cells treated with MX, accompanying with an increase in cyt-c excretion, but caspase 8 expression is unaffected (Fig. 6). These findings demonstrate that the *FANCF* silencing-induced apoptosis is mediated by the mitochondrial pathway, rather than the death receptor pathway. Therefore, enhanced MX sensitivity in *FANCF*-silenced MCF-7 and T-47D cells may partly due to the regulation of the mitochondrial apoptosis pathway.

Two cell lines MCF-7 and T-47D increase the sensitivity to MX when *FANCF* is silenced, and a greater effect is found in MCF-7 cells than that in T-47D cells. The question arise as to what are the mechanisms underlying the different response to MX after *FANCF* silencing. We find that mitochondria-mediated apoptosis, BCRP inhibition, p38 and JNK activation in *FANCF*-silenced MCF-7 cells are stronger than those in *FANCF*-silenced T-47D cells. These findings agree with the greater MX sensitivity in *FANCF*-silenced MCF-7 cells. Additionally, several studies have shown that the DNA damage induced by topo II inhibitor lead to p53-dependent cell cycle arrest or apoptosis [45], [46]. Wild-type *p53*, but not mutant *p53*, is capable of inhibiting transcription of the MDR1 gene and BCRP, thereby increasing the drug sensitivity of tumor cells [47], [48]. We find that treatment with *FANCF* shRNA and MX results in an increase of p53 expression, suggesting that p53 pathway are likely important determinants of *FANCF*-silenced breast cancer cells sensitivity to MX. However, other factors, such as Her-2 levels, casapase-3 status, and bcl-2 status (Table 1) and so on may contribute to the different MX sensitivity induced by *FANCF* silencing in the two cell lines. Future studies using p53 RNAi and p53 knockout cells will be performed to identify the roles of p53 in *FANCF*-mediated potentiation of the sensitivity to MX in breast cancer cells.

Recent study shows that FANCF deficient mice develop spontaneous tumors, and show aberrant response to DNA cross-linking agents, suggesting that FANCF is critical in the FA/BRCA pathway for repairing of DNA double-strand breaks induced by DNA cross-linking agents [49]. It is well known that MX induces crosslinks and double-strand breaks (DSBs) in DNA [50], and FA/BRCA pathway plays an important role in DNA repair [51]. In FANCF-deficient cancer cells, the repair of double-strand DNA break by homologous recombination is damaged, thus rendering the cancer cells highly sensitive to alternative double-strand break repair (DSBR) pathways, such as nonhomologous end-joining (NHEJ) single-strand annealing (SSA) [52]. Therefore, blockade of the alternative DSBR pathway in the FANCF-deficient cancers will indicate a synthetic lethal treatment for the *FANCF*-deficient cancers. In this study, we find that knockdown of *FANCF* potentiates the sensitivity of breast cancer cells to MX. This could results from the synthetic lethal interaction of MX with the DSBR pathway. Thus, MX can be more toxic to *FANCF*-deficient cancers cells compared to healthy cells, since *FANCF*-deficient cancers lack the necessary DNA repair pathways that promote survival in healthy cells.

In summary, our findings suggest a new therapeutic strategy for breast cancer. *FANCF* silencing-induced dysfunction of the FA/BRCA pathway increases sensitivity of human breast cancer cell line to MX, possibly by activating the JNK, p38 MAPK signaling pathway, subsequently increasing p53 activity, activating mitochondrial apoptosis pathway, and decreasing BCRP expression. FANCF might be a candidate target to develop novel therapeutic strategy to enhance response to topoisomerase II poisons in breast cancer.
